# Bolaamphiphilic properties and pH-dependent micellization of quercetin polyglycoside†

**DOI:** 10.1039/c9ra05711k

**Published:** 2019-10-21

**Authors:** Mahmuda Nargis, Abu Bin Ihsan, Yasuhito Koyama

**Affiliations:** Department of Pharmaceutical Engineering, Faculty of Engineering, Toyama Prefectural University 5180 Kurokawa Imizu Toyama 939-0398 Japan ykoyama@pu-toyama.ac.jp

## Abstract

Quercetin polyglycoside as a new bolaamphiphile is prepared *via* a one-pot grafting polymerization technique using sugar-based cyclic sulfite. Micelles comprising quercetin polyglycoside exhibit special pH-effects, in which the polyglycoside moieties on the surface of the micelle serve as a steric protecting group to endow chemical stabilization.

## Abstract 2

Micelles comprising quercetin polyglycoside skeletons exhibit special pH-effects based on the integration of bulky polyglycosides on the surface.

## Graphical abstract

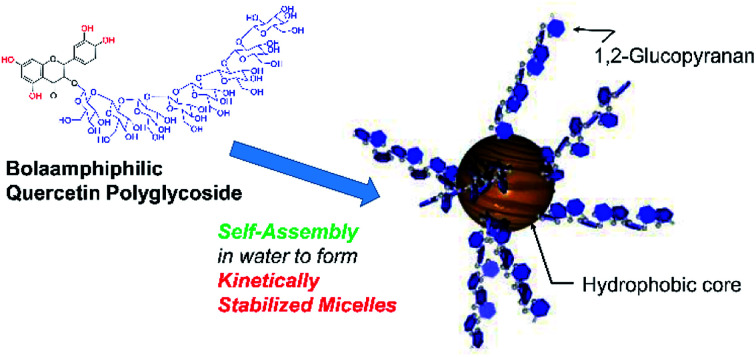

Quercetin glycosides (QGs) constitute a fascinating class of flavonoid glycosides,^1^ where quercetin as a phenolic aglycon covalently binds to sugars *via* glycoside linkages. QGs are included in several fruits and vegetables as an ingredient and show high solubility in water. When QGs are ingested, chewing and digestion processes facilitate the hydrolysis of glycon to generate hydrophobic quercetin. It has been reported that quercetin works as the actual bioactive site of QGs and exhibits various physiological effects.^2^QGs can be regarded as a bolaamphiphilic compound that comprises polar groups at both ends of a hydrophobe.^3^ Bolaamphiphiles have potential usefulness as a building block for well-organized aggregates in water.^4^ However, the structural correlation of QGs to self-assemble in water hasn't been investigated to date. The micelles consisting of QGs are also expected to show pH-responsivity due to the acidic phenols on the aglycon part.

On the other hand, we have recently reported a new synthetic method of glycoside grafting by a (1→2)-glucopyranan skeleton.^5^ To design new bolaamphiphilic QG derivatives and evaluate the effects of glycoside on the micellization behaviors of quercetin, we planned to adopt our invented technique to quercetin skeleton, considering the structures of natural quercetin derivatives bearing (1→2)-glucopyranan at the 3 position such as rutin^6^ and quercetin 3-*O*-sophoroside.^7^ Herein, we describe the synthesis and micellization of quercetin polyglycoside. We systematically studied the bolaamphiphilic properties of quercetin polyglycoside. Quercetin polyglycoside forms micelles in aqueous media with a wide range of pH values from pH 4.0 to 10.0, while quercetin hardly forms micelles in pH 10.0 aqueous medium. It's amazing to observe that the critical micelle concentration (CMC) values of both quercetin polyglycoside and quercetin become smaller along the increase in the pH value, which is completely opposite to the normal tendency of micellization of typical amphiphilic compounds. We termed such micellar behaviors as special pH-effects. It is indicated that the unusual CMC tendency would be attributed to the acid dissociation equilibrium of polyphenolic quercetin skeleton. We discuss the effects of polyglycoside on the stabilization of micelle from the viewpoint of steric protecting group for reactive species.

Aglycon 1 and sugar-based cyclic sulfite 2 were prepared according to the literatures (Scheme 1).^8,9^ The cationic ring-opening polycondensation of 2 was initiated by 1 with a catalytic amount of trifluoromethanesulfonic acid (TfOH) in the presence of molecular sieves 3 Å (MS 3 A) in CH_2_Cl_2_ at room temperature. The reaction mixture was stirred for 13 d. After typical workup, the crude material was purified by a gel permeation chromatography (GPC) to give the polymer 3. The structure of 3 was confirmed by IR, ^13^C NMR, and ^1^H NMR spectra.^10^ The molecular weight of 3 and its distribution were estimated by ^1^H NMR spectrum and a size exclusion column chromatography (SEC). In the IR spectrum of 3, we observed the characteristic carbonyl absorption signal at 1728 cm^−1^,^10^ indicating the presence of aglycon structure in the polymer framework. In addition, we confirmed the absence of absorption signal from sulfite linkage at around 1200 cm^−1^,^10^ suggesting that the polymerization accompanied with the clean elimination of SO_2_ from the main chain to give 1,2-glycosidic skeleton.^5^ The α/β ratio in the anomeric stereocenters of 3 was almost 1 : 1, which was estimated by the ^13^C NMR spectrum using the integral ratio between the carbon signals at 110–100 ppm for β carbons and those at 100–90 ppm for α carbons.

**Scheme 1:**
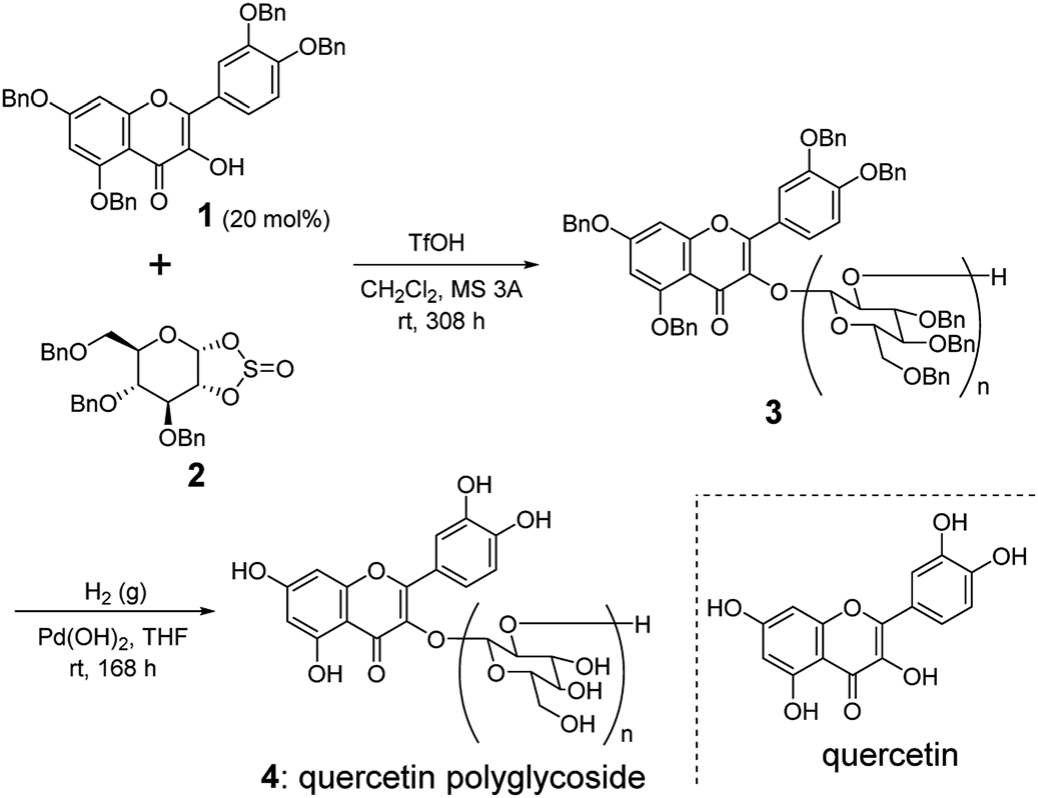
Synthesis of quercetin polyglycoside 4*via* ring-opening polycondensation of sugar-based cyclic sulfite 2.

From the integral ratio between the aromatic and aliphatic proton signals in the ^1^H NMR spectrum, we estimated the degree of polymerization (DP) and *M*_n_ to be 9.2 and 4.6 kDa, respectively. The obtained DP value of 3 was slightly higher than the expected value from the feed ratio of 1 to 2, which indicates the low initiator efficiency of 1 on the polymerization. The alcohol of 1 would be sterically hindered due to the proximity of biaryl linkage, which could slowly initiate the addition reaction rate of 1 to 2 to give the corresponding monoglycoside with a free alcohol at the 2 position of the glycon. The resultant secondary alcohol on the monoglycoside might have a higher reactivity to 2 rather than the alcohol of 1, which could lead to higher DP than that expected from the feed ratio. The polydispersity index (*M*_w_/*M*_n_) of 3 was estimated by SEC to be 1.2.

We next performed the hydrogenolysis of 3 using Pd(OH)_2_ as a heterogeneous catalyst under H_2_ atmosphere. After 7 d, the reaction mixture was filtered and concentrated *in vacuo* to afford quercetin polyglycoside (4) in a quantitative yield. The structure of 4 was confirmed by the ^1^H NMR and IR spectra.^10^ Polyglycoside 4 exhibited high solubility in water.

Fig. 1a shows the UV-vis spectra of quercetin and 4 in pH 7.0 aqueous media. The absorption spectrum of quercetin exhibits a maximum at around 380 nm, while that of 4 is not clear. The molar absorbance of 4 at longer wavelength region is smaller than that of quercetin.

**Fig. 1:**
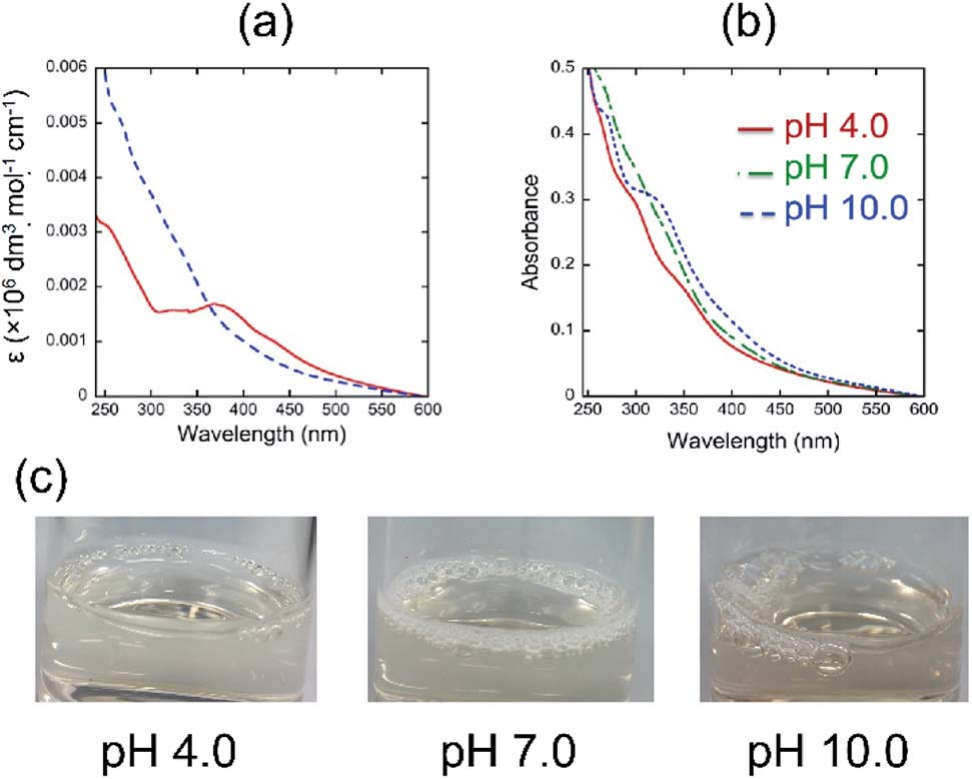
(a) UV-vis spectra of quercetin (red solid line) and 4 (blue dotted line) in pH 7.0 aqueous media (0.02 wt%) at 25 °C, (b) UV-vis spectra of 4 in pH 4.0 (red), pH 7.0 (green), and pH 10.0 (blue) aqueous media (0.02 wt%) at 25 °C, and (c) foam formation of 4.

The results indicate that quercetin has a longer effective conjugation length than that of 4, suggesting that the introduction of polyglycoside to the 3 position of quercetin might make the biaryl linkage twisted, which could decrease the effective conjugation length. We next measured the UV-vis spectra of 4 in aqueous media with different pH values (Fig. 1b). It was found that the absorbance of 4 at longer wavelength region becomes more intense as the pH value in the solution of 4 is higher. The UV-vis spectrum of 4 in pH 10.0 includes an absorption maximum at around 340 nm. Considering the p*K*_a_ values of typical phenolic compounds, we concluded that 4 forms phenoxide in the pH 10.0 aqueous medium. On the contrary, the spectrum of 4 in the pH 4.0 aqueous medium should mean the spectrum of fully protonated species bearing neutral phenols. The spectrum of 4 in pH 7.0 appears at the middle position between that in pH 4.0 and in pH 10.0, indicating the partial deprotonation of phenols. The pH-dependency of spectral shape of 4 is in a good accordance with that of quercetin.^10^ In visual observation, we noticed that the highly concentrated aqueous solutions of 4 have strong foam-forming tendency (Fig. 1c), which is in a good agreement with our expectation that it can form micelles. To confirm the micellization of 4, we measured the UV-vis spectra of aqueous solutions of 4 at various concentrations to know these CMC values, *i.e*., onset concentration to form micelles, according to the literature.^11^ The absorbance values at 354 nm were normalized by the concentration and plotted against the sample concentration (Fig. 2). All figures clearly include an intersection point as the CMC of 4, strongly supporting the formation of micelles. As a reference, we also determined the CMC values of quercetin at the similar manner.^10^ The CMC values of 4 and quercetin were summarized in Table 1.

**Fig. 2:**
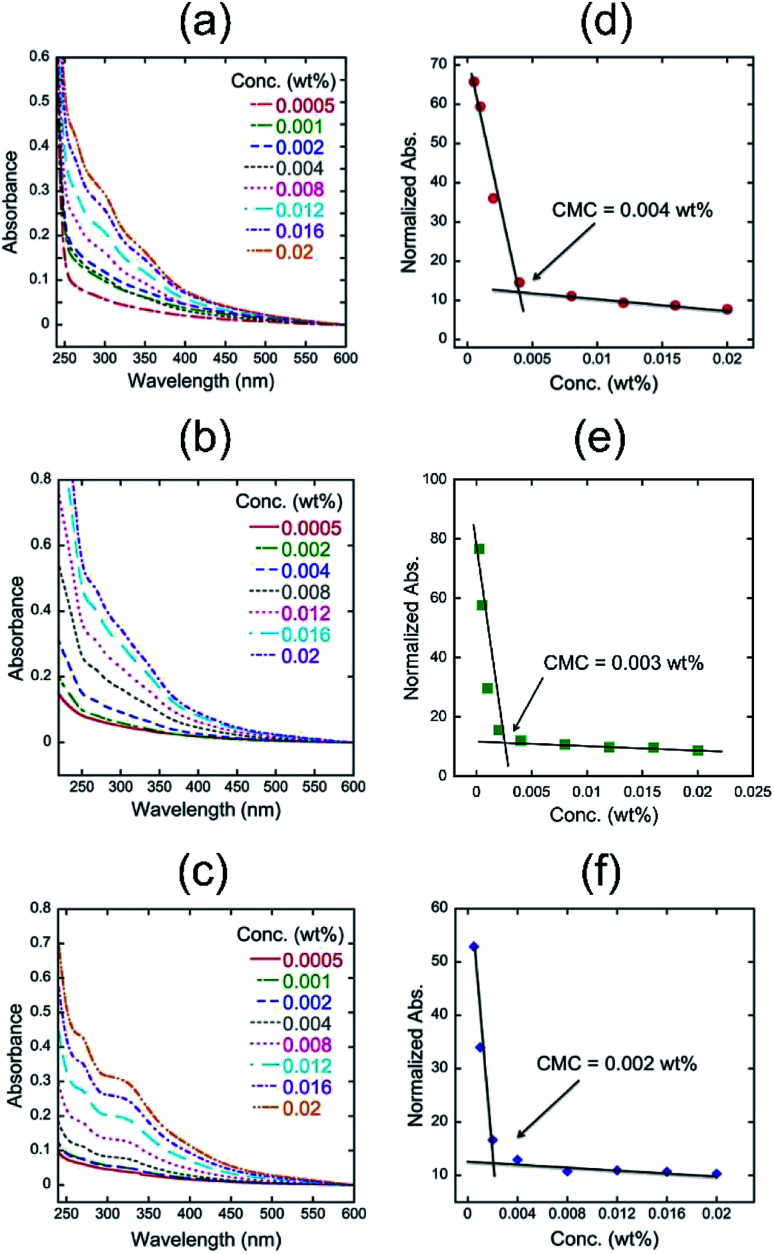
UV-vis spectra of 4 at 25 °C in (a) pH 4.0, (b) pH 7.0, and (c) pH 10.0 aqueous media and normalized absorbance of 4 at 354 nm as a function of concentration (wt%) in (d) pH 4.0, (e) pH 7.0, and (f) pH 10.0 aqueous media.

**Table 1:** Effects of pH on the CMC values of 4 and quercetin

	CMC (wt%) in pH 4.0	CMC (wt%) in pH 7.0	CMC (wt%) in pH 10.0
4	4 × 10^−3^	3 × 10^−3^	2 × 10^−3^
Quercetin	9 × 10^−4^	7 × 10^−4^	Not observed

The CMC values of 4 in pH 4.0 and 7.0 were approximately five times higher than those of quercetin, which could be ascribed to both the difference of molecular weight and the increased hydrophilicity of 4. It is interesting that the CMC values of 4 become smaller along with the increase in the pH value, despite of the formation of hydrophilic phenoxide in basic media. The similar tendency is also observed in the CMC values of quercetin. The results make us to hypothesize that such pH-dependence of CMC could be attributed to the acid dissociation equilibrium of quercetin structure. The partial deprotonation of phenols in quercetin structure might facilitate intermolecular hydrogen bonding between phenoxide and neutral phenol, leading to the effective formation of associates. It is highlighted that no CMC was observed in the pH 10.0 solution of quercetin, indicating that quercetin hardly forms micelles in the basic medium. The strong ionic repulsion between phenoxides would suppress the formation of associates. On the other hand, the aqueous solution of 4 even in pH 10.0 was found to form micelles, suggesting the chemical stability of micelles comprising 4.

Fig. 3 shows the UV-vis spectra of quercetin and 4 in pH 10.0 aqueous media above CMC (0.02 wt%). While quercetin exhibits intense molar absorption at around 340 nm, the absorption of 4 was almost silent at the wavelength region. Such remarkable hypochromic effect on 4 would suggest the proximity of aglycon with that of the other molecule in the micelles. The bulky polyglycoside moiety of 4 appears to serve as a steric protecting group for the stabilization of micelle (Fig. 4). The bulky polyglycosides should be densely integrated on the surface of micelle, which would prevent the approach of small molecules such as water and hydroxide to the hydrophobic core. Such stabilization effects of micelles comprising 4 seems to be corresponding to the concept of kinetic stabilization for low-molecular-weight chemical species with high reactivity.^12^ The presence of bulky polyglycosides on the surface of micelle contributes to the kinetic stability of micelle comprising 4, which can suppress the dissociation of micelles in the basic environment.

**Fig. 3:**
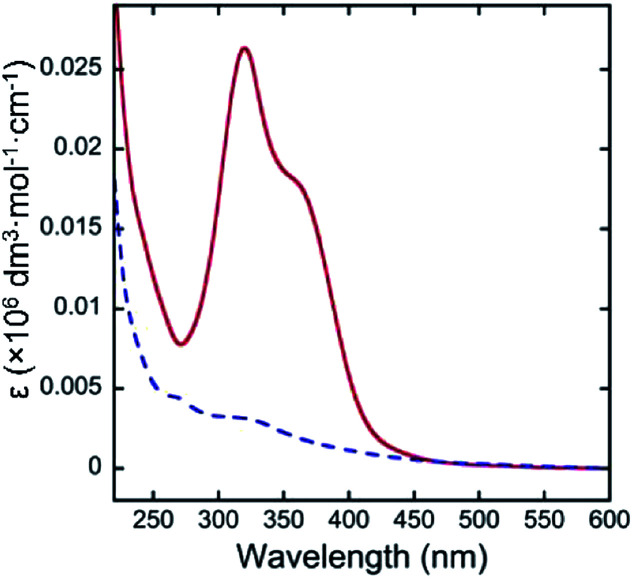
UV-vis spectra of quercetin (red solid line) and 4 (blue dotted line) in pH 10.0 aqueous media above CMC (0.02 wt%) at 25 °C.

**Fig. 4:**
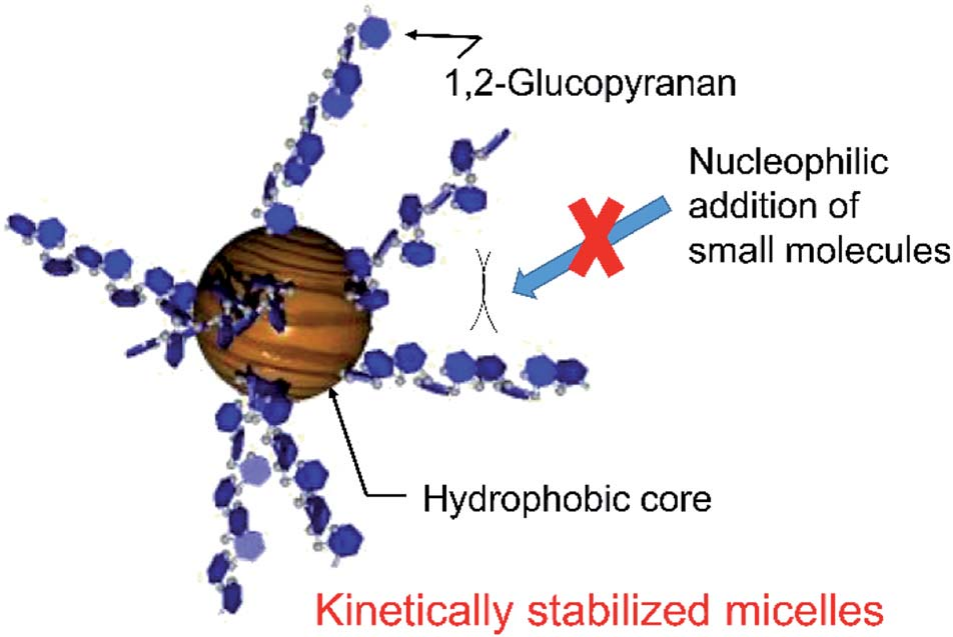
Plausible structure for the kinetically stabilized micelle of 4.

In conclusion, we prepared quercetin polyglycoside as a new bolaamphiphile *via* one-pot grafting polymerization using sugar-based cyclic sulfite 2 as a monomer. We investigated the effects of polyglycoside on the micellization of quercetin by using the solutions of quercetin polyglycoside 4 and quercetin in aqueous media with different pH values. The polyphenol structure of quercetin exhibits unique pH-dependency of CMC that becomes smaller as pH value increased. While quercetin hardly forms micelles in pH 10.0 aqueous medium, the solution of 4 forms micelles in the medium, indicating the special role of polyglycoside on the stabilization of micelle. This is the first report on the self-assemble behaviors of quercetin and its glycoside. The present study may open a new insight into the members of flavonoid glycosides as a bolaamphiphile. It is noted that the CMC value of 4 in pH 4.0 aqueous medium appears to be higher than those in higher pH media. The micelles of 4 prepared at an appropriate concentration are expected to dissociate at acidic environment in various diseased conditions. Although this work has focused on the evaluation of fundamental micellization behaviors of QGs, such pH-responsive CMC values and bolaamphiphilic nature of 4 would indicate the application to stimuli-responsive micelles as a drug carrier that enables controlled release of drugs.^13^ The special pH-effects observed in this work will certainly motivates supramolecular chemist and synthetic biologist to adopt natural glycoside derivatives to micellar chemistry. The underlying mechanism of micellization and detailed physical properties of the micelles are currently investigating.

## Conflicts of interest

There are no conflicts to declare.

## Supplementary Material

RA-009-C9RA05711K-s001
