# Supplementary material for: Bolaamphiphilic properties and pH-dependent micellization of quercetin polyglycoside
Source: RSC Adv. 2019 Oct 21;9(58):33674–7. doi: 10.1039/c9ra05711k (PMC9073524; doi:10.1039/c9ra05711k)
Supplement: RA-009-C9RA05711K-s001 [file RA-009-C9RA05711K-s001.pdf]

Electronic Supplementary Material (ESI) for RSC Advances.
This journal is © The Royal Society of Chemistry 2019

Supplementary Materials

Bolaamphiphilic Properties and pH-Dependent Micellization of Quercetin Polyglycoside

Mahmuda Nargis, Abu Bin Ihsan, and Yasuhito Koyama*

Department of Pharmaceutical Engineering, Faculty of Engineering
Toyama Prefectural University, 5180 Kurokawa, Imizu, Toyama 939-0398, Japan

*E-mail: ykoyama@pu-toyama.ac.jp

Contents

General methods **2**

Typical procedure for the polymerization of cyclic sulfite **3**

Typical procedure for the deprotection of benzyl group **4**

^{1}H NMR, ^{13}C NMR, and IR spectra **5**

UV-vis spectra and CMC plots of quercetin **8**

Plots of absorbance as a function of logarithm of concentration **9**

Dependence of pH on the CMC values **10**

DLS profiles **10**

References **12**

General methods.

Materials: Dehydrated dichloromethane (Kanto Chemical Co., Inc., Tokyo, Japan), trifluoromethanesulfonic acid (TfOH, Kanto Chemical Co., Inc. , Tokyo, Japan), 50 wt% $Pd(OH)_2$ (Tokyo Chemical Industry Co., Inc., Tokyo, Japan), MeOH (Taiyo Chemicals, Co., Ltd., Wakayama, Japan), pH 7.0 buffer solution (KH_2PO_4-NaOH, Tokyo Chemical Industry Co., Ltd., Tokyo, Japan), and pH 10.0 buffer solution ($NaHCO_3$-NaOH, Tokyo Chemical Industry Co., Ltd., Tokyo, Japan) were used without further purification. Granular molecular sieves 3Å (MS 3A) as a dehydrator was activated by careful heating by a heat gun under vacuum. The other chemicals were used without purification. Tetrabenzylated quercetin **1** was prepared according to the reported method with a slight modification: M. D. L. de la Torre, A. G. P. Rodrigues, A. C. Tomé, A. M. S. Silva, J. A. S. Cavaleiro, *Tetrahedron*, **2004**, *60*, 3581. Sugar-based cyclic sulfite was prepared according to the literature: S. S. Sangeetha, Y. Koyama, *Tetrahedron Lett.,* **2016**, *57*, 3657. The pH 4.0 buffer solution was prepared by citric acid and Na_2HPO_4 according to a typical procedure for McIlvaine buffer: T. C. McIlvaine, *J. Biol. Chem.* **1921**, *49*, 183.

Measurements: ^{1}H NMR (400 MHz) and ^{13}C NMR (100 MHz) spectra were recorded on Bruker AVANCE II 400 spectrometers (Brucker, Fällanden, Switzerland) using $CDCl_3$, DMSO-d_6, and CD_3OD as the solvent, calibrated using an internal standard and residual undeuterated solvent signals. In the cases of ^{1}H NMR spectra in DMSO-d_6, a small amount of CF_3COOH was added to the NMR sample in order to shift the water signal. FT-IR spectra via an attenuated total reflection (ATR) method were measured using a Perkin Elmer spectrum 100 spectrometer (Perkin Elmer, Shelton, USA). UV–vis spectra were recorded on a JASCO V-550 spectrophotometer (JASCO Co. Ltd., Tokyo, Japan) using a 1.0 cm path quartz cell with a temperature controller (ETC-505T, JASCO Co. Ltd., Tokyo, Japan). The size distributions of the micelles were measured with a dynamic light

scattering (DLS) instrument (Zetasizer nano ZSP, Malvern Co. Ltd.). The time-dependent correlation function of the scattered light intensity was measured at a scattering angle of 173°. The size distributions were determined using the software provided with the instrument.

Determination of critical micelle concentration (CMC).

Stock solutions of the samples were prepared by dissolving in aqueous buffer solutions. The aqueous solutions of various concentrations were prepared using the stock solution by proper dilution. The UV–vis spectra of the sample solutions were recorded on a JASCO V-550 spectrophotometer (JASCO Co. Ltd., Tokyo, Japan) using a 1.0 cm path quartz cell at 25 °C. The obtained absorbance values at 354 nm were standardized by the concentration, which were plotted as the function of concentration (Figures 2 and S6).[1] From the intersection point in the plots, we determined the CMC values of the polymers in water. In addition, we also plotted the obtained absorbance values at 354 nm as a function of logarithm of concentration according to the literatures using the other method for CMC determination (Figures S7 and S8).[2-8] The intersection points in the plots indicate the same CMC values as those obtained by Figures 2 and S6.

Typical procedure for the polymerization of cyclic sulfite.

To a solution of cyclic sulfite **2** (119.2 mg, 0.240 mmol) and initiator **1** (31.9 mg, 0.048 mmol) in CH_2Cl_2 (0.80 mL) was added a freshly activated MS 3A (ca. 29 mg) as a dehydrator at room temperature. After stirring for 1 h at room temperature, the mixture was cooled to 0 °C. TfOH (4.3 μL, 0.048 mmol) was added to the mixture. The mixture was warmed to room temperature and stirred for 212 h at the same temperature. Additional TfOH (4.3 μL, 0.048 mmol) was added to the mixture. The mixture was further stirred for 96 h. The reaction was quenched by the addition of Et_3N (50 μL) and sat. aq. $NaHCO_3$. The products were extracted with $CHCl_3$, repeatedly. The

combined organic layer was dried over $MgSO_4$, filtered, and concentrated in vacuo to give a crude material (140 mg, quant.). The crude material was purified by gel permeation chromatography (GPC, eluent: $CHCl_3$) to give the polymer **3** (76.8 mg, 57%) as an analytical pure sample. The degree of polymerization (DP) and number average molecular weight (M_n) were estimated by integral ratio between the aromatic proton signals and the aliphatic protons in the ^{1}H NMR spectrum (DMSO-d_6) to be DP 9.2 and M_n 4,600 Da. The polydispersity index (M_w/M_n) was also estimated by a size exclusion column chromatography (SEC, eluent: THF) on the basis of polystyrene standards to be M_w/M_n 1.2: ^{1}H NMR (400 MHz, 298 K, DMSO-d_6) δ 7.60–6.80 (m, 163H), 5.40-3.10 (m, 127.6H) ppm; ^{13}C NMR (100 MHz, 298 K, $CDCl_3$) δ 140-138 (brd), 128-126 (m), 110-90 (m), 84-68 (m); IR (ATR) υ 3458, 3064, 3031, 2920, 2865, 1728, 1605, 1497, 1454, 1361, 1264, 1209, 1047, 1026, 912, 732, 695 cm^{-1}.

Typical procedure for the deprotection of benzyl group.

The solution of benzylated polymer **3** (72.6 mg) in THF (3.0 mL) was added to the suspension of $Pd(OH)_2$ (50 wt%, 72 mg) in water (1.0 mL). The reaction system was degassed 3 times using vacuum pump and the atmosphere was replaced to H_2. The mixture was stirred for 168 h at room temperature under H_2 atmosphere. The reaction mixture was filtered through a celite pad and the cake was repeatedly washed with MeOH. The filtrate was concentrated in vacuo and dried under vacuum to give the deprotected polymer **4** (39.7 mg) in a quantitative yield; ^{1}H NMR (400 MHz, 298 K, MeOD) δ 8.10–7.10 (m), 5.40-4.40 (m), 4.10-3.10 (m) ppm; IR (ATR) υ 3308, 2928, 1728, 1635, 1360, 1258, 1016, 918 cm^{-1}.

^{1}H NMR, ^{13}C NMR, and IR spectra

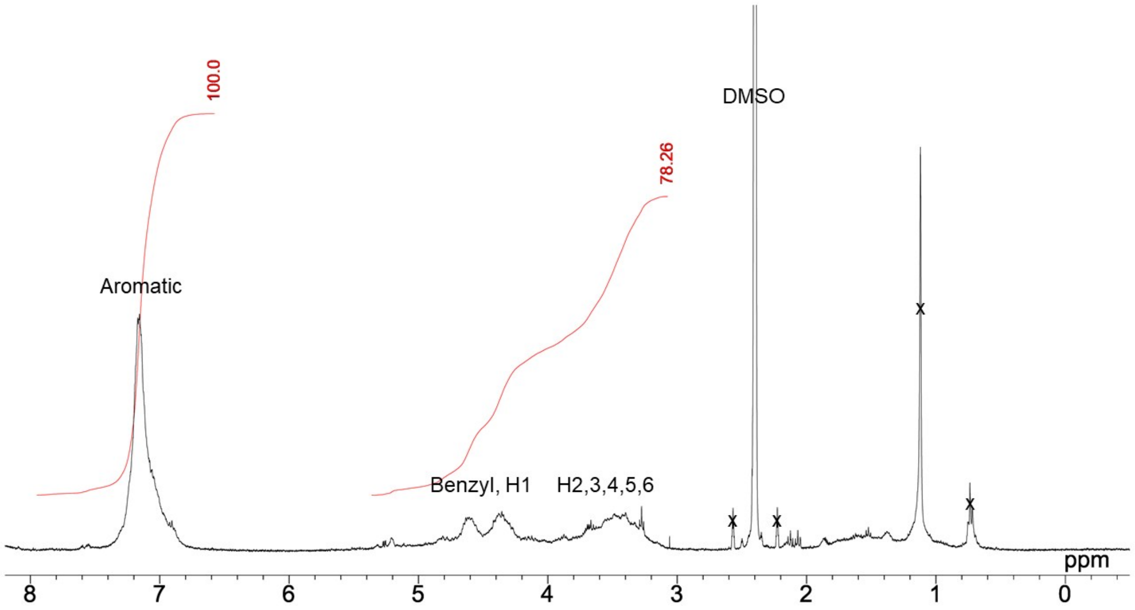

Figure S1: ^{1}H NMR spectrum of **3** (400 MHz, DMSO-d_6 with a small amount of CF_3COOH, 298 K).

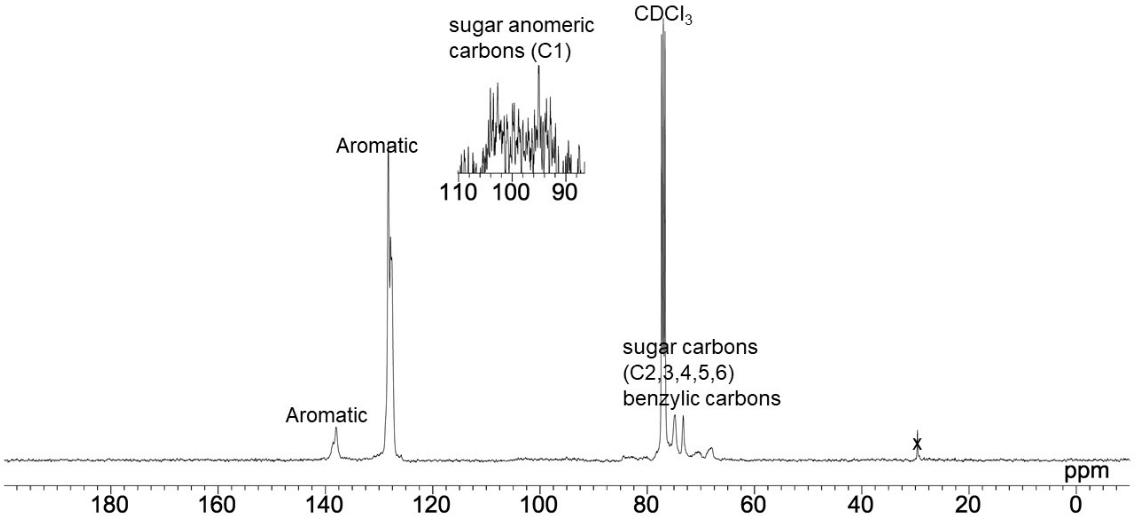

Figure S2: ^{13}C NMR spectrum of **3** (100 MHz, $CDCl_3$, 298 K) and the inset of expanded anomeric carbon region.

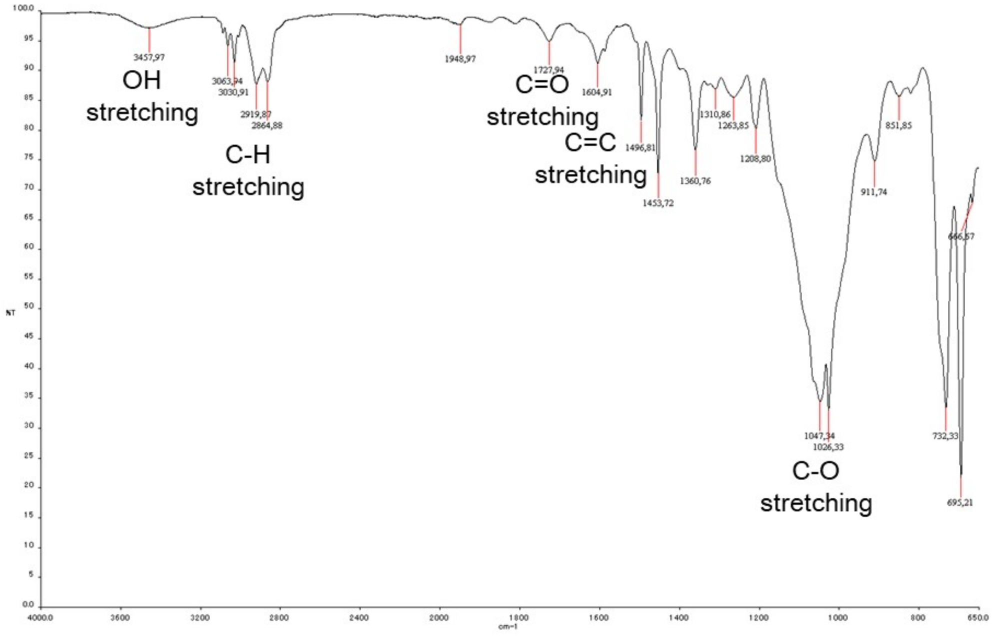

Figure S3. IR spectrum of **3** (ATR).

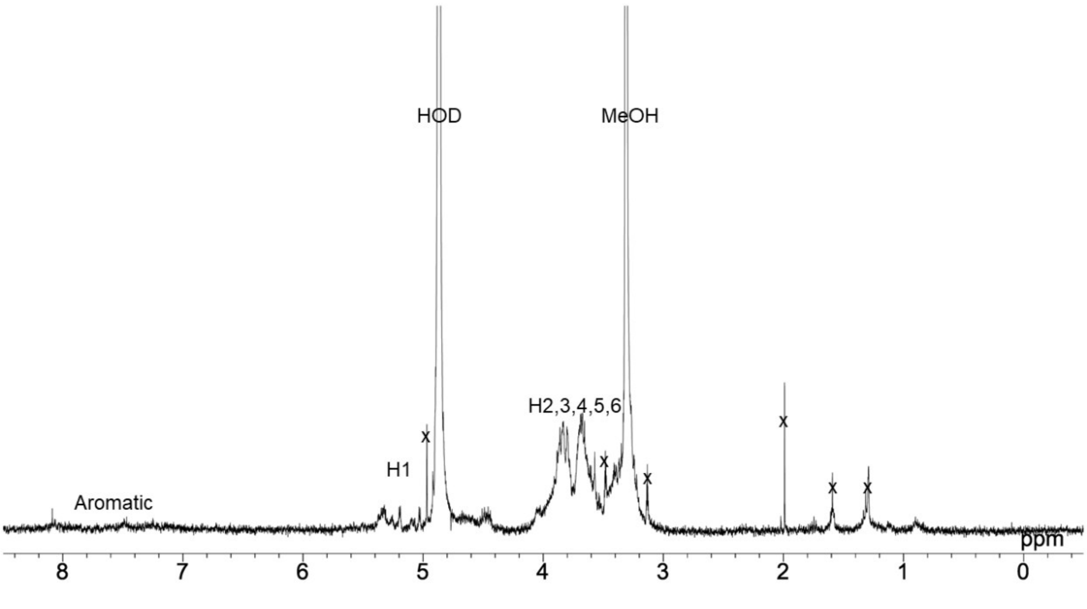

Figure S4: ^{1}H NMR spectrum of **4** (400 MHz, CD_3OD, 298 K).

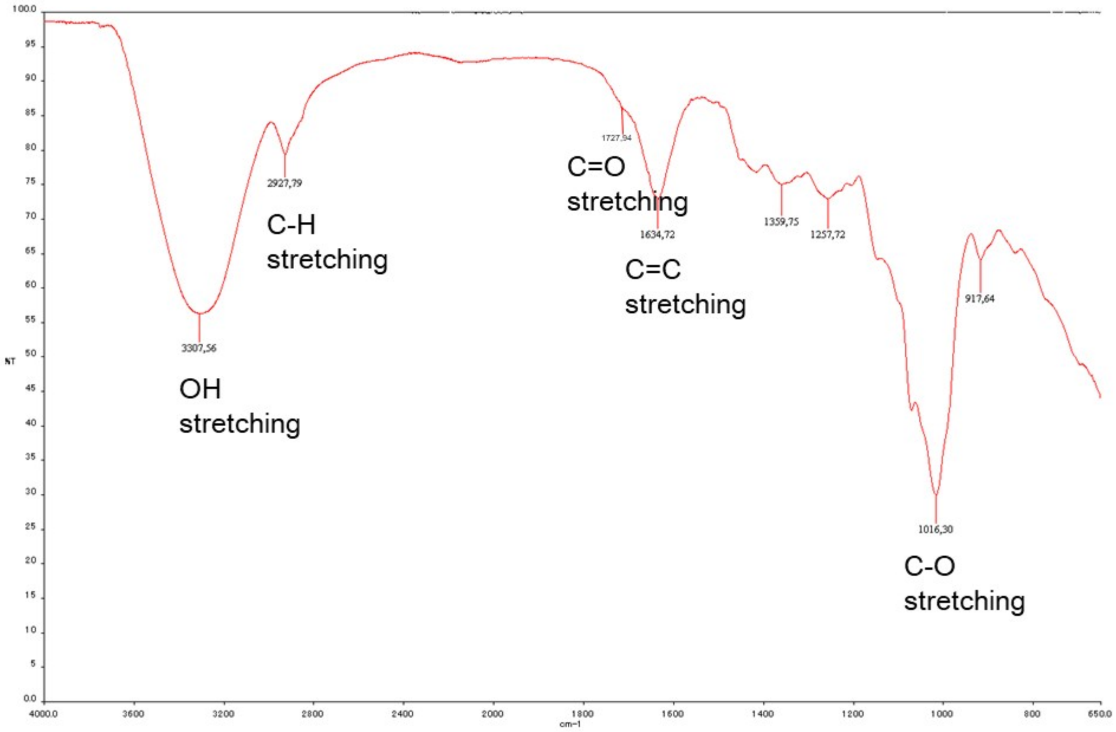

Figure S5. IR spectrum of **4** (ATR).

UV-vis spectra and CMC plots of quercetin

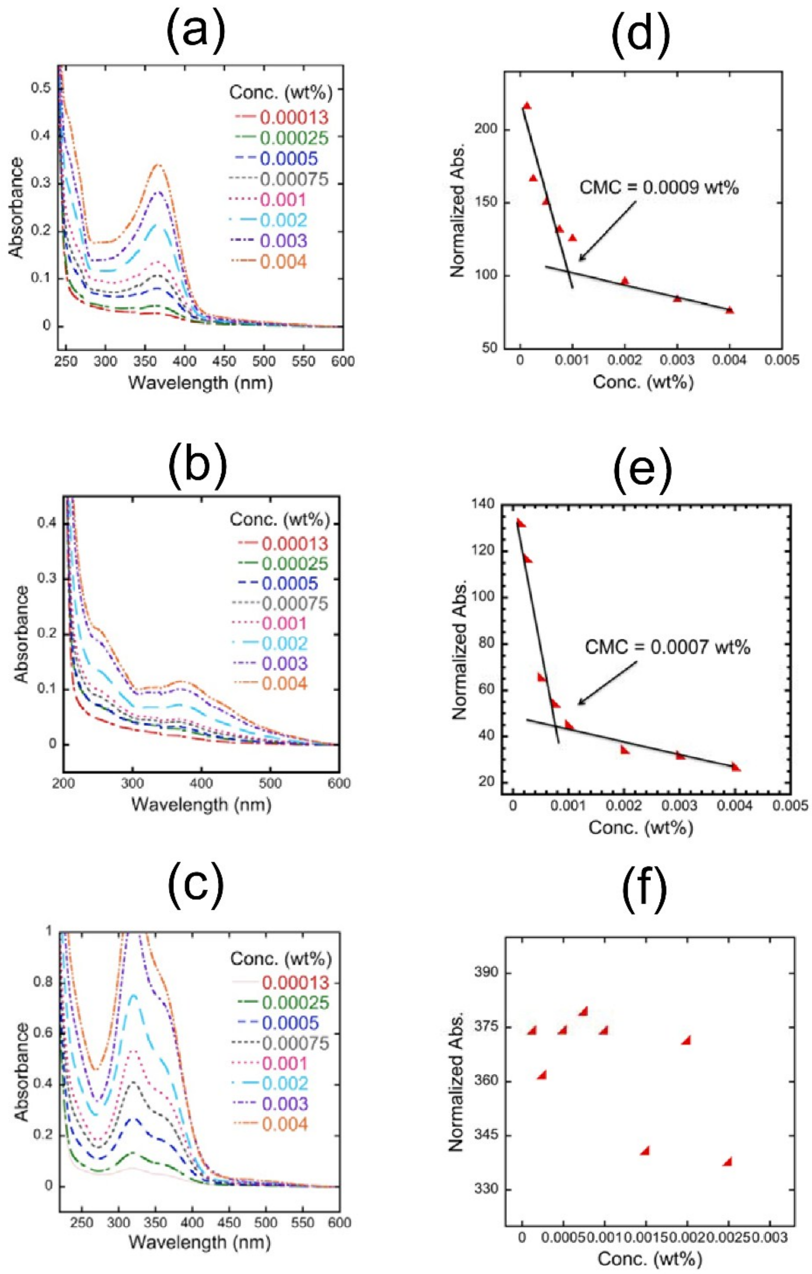

Figure S6. UV-vis spectra of quercetin at 25 °C in (a) pH 4.0, (b) pH 7.0, and (c) pH 10.0 aqueous media and normalized absorbance of quercetin at 354 nm as a function of concentration (wt%) in (d) pH 4.0, (e) pH 7.0, and (f) pH 10.0 aqueous media.

Plots of absorbance as a function of logarithm of concentration[2-7]

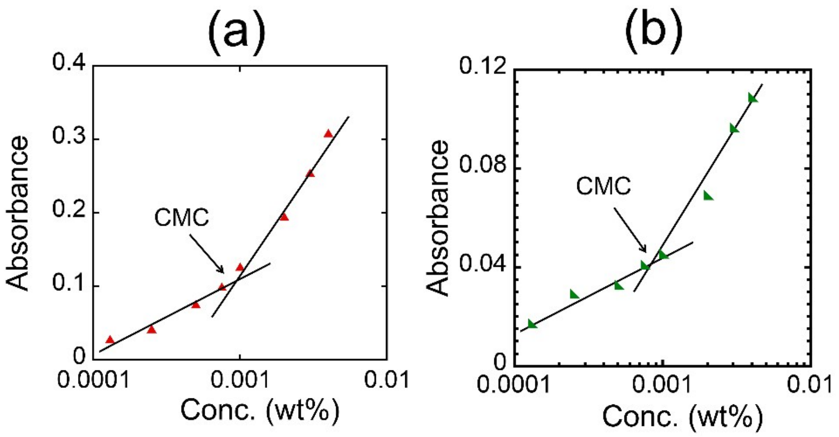

Figure S7. The absorbance of quercetin in (a) pH 4.0 and (b) pH 7.0 aqueous media at 354 nm as a function of logarithm of concentration (wt%) at 25 °C.

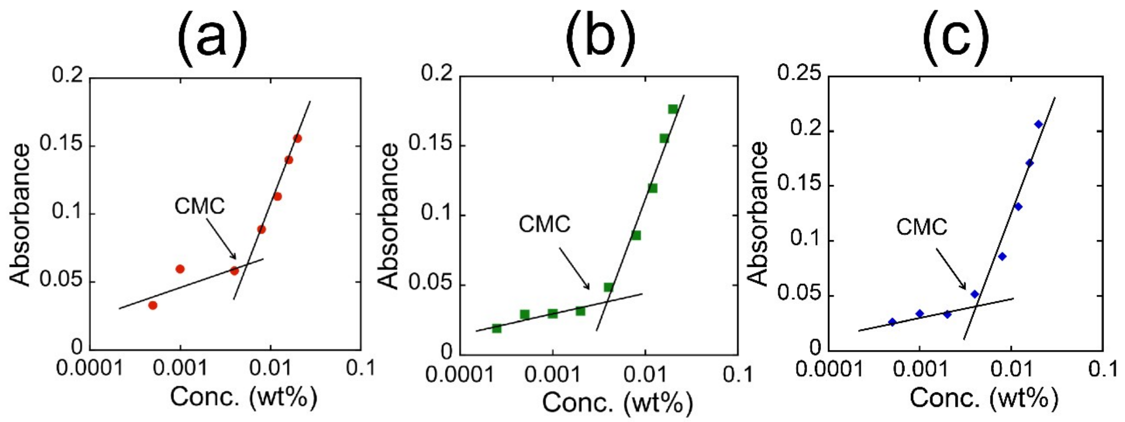

Figure S8. The absorbance of **4** in (a) pH 4.0, (b) pH 7.0, and (c) pH 10.0 aqueous media at 354 nm as a function of logarithm of concentration (wt%) at 25 °C.

Dependence of pH on the CMC values

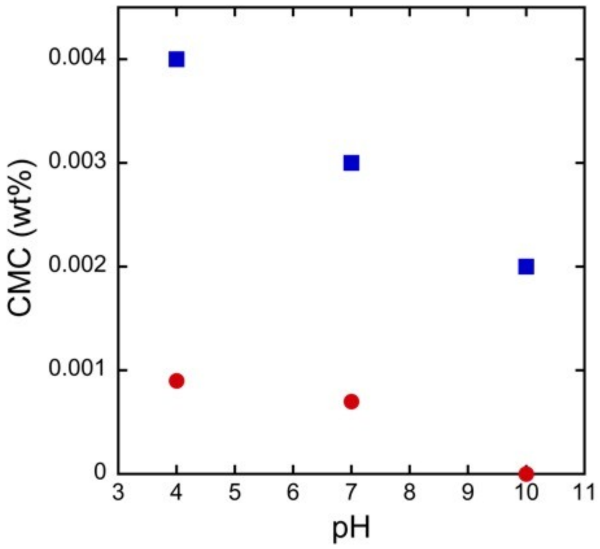

Figure S9. Dependence of pH on the CMC values of quercetin (red circle) and **4** (blue square).

DLS profiles

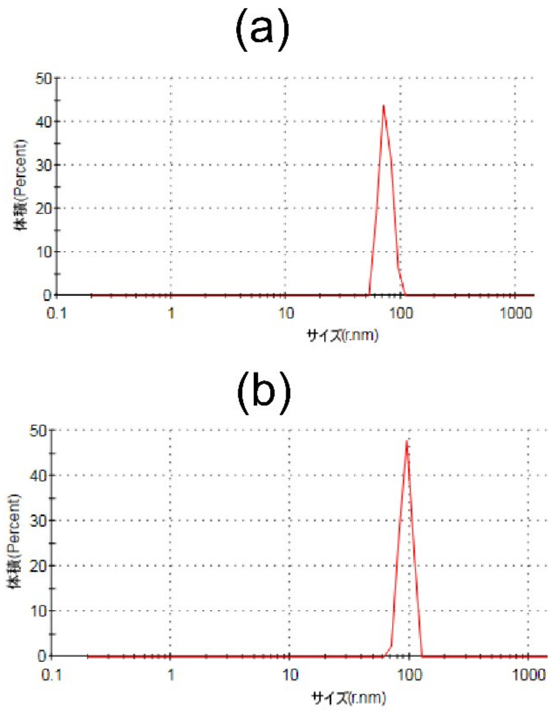

Figure S10. Volume *vs.* size for calculating hydrodynamic size (nm) of quercetin obtained from dynamic light scattering (DLS) measurements in (a) pH 4.0 and (b) pH 7.0 aqueous media at 25 °C above the CMC.

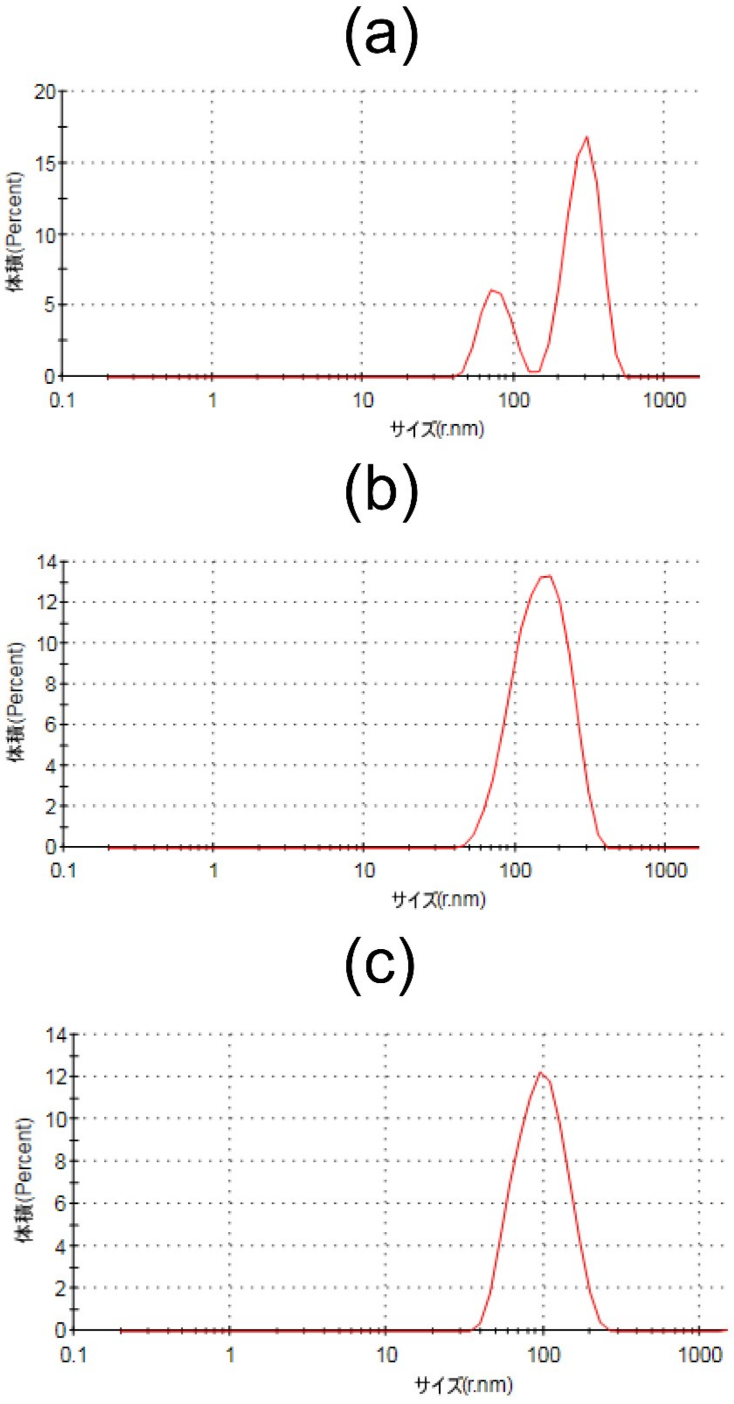

Figure S11. Volume *vs.* size for calculating hydrodynamic size (nm) of **4** obtained from dynamic light scattering (DLS) measurements in (a) pH 4.0, (b) pH 7.0, and (c) pH 10.0 aqueous media at 25 °C above the CMC.

Table S1. Results of DLS profiles at 25 °C above the CMC.

	pH	Hydrodynamic radius of micelle (nm)
Quercetin	4.0	74
	7.0	94
	10.0	No micelle formed
**4**	4.0	294, 78 (bimodal)
	7.0	158
	10.0	102

References

1. Mukerjee, P., In *Natural Standard Reference Data System, Natural Bureau of Standards*, U.S. Department of Commerce Ed. U.S. Government Printing Office, Washington, D.C., U.S.A., 1971, pp. 1-222.
2. Cai, L.; Gochin, M.; Liu, K. A facile surfactant critical micelle concentration determination. *Chem. Commun.* **2011**, *47*, 5527–5529.
3. Ihsan, A. B.; Nargis, M.; Koyama, Y. Effects of the hydrophilic-lipophilic balance of alternating peptides on self-assembly and thermo-responsive behaviors. *Int. J. Mol. Sci.* **2019**, *20*, 4604/1-11.
4. Nesměrák, K.; Němcová, I. Determination of critical micelle concentration by electrochemical means. *Anal. Lett.* **2006**, *39*, 1023–1040.
5. Li, N.; Luo, H.; Liu, S. A new method for the determination of the critical micelle concentration of Triton X-100 in the absence and presence of β-cyclodextrn by resonance Rayleigh scattering technology. *Spectrochim. Acta Part A Mol. Biomol. Spectrosc.* **2004**, *60*, 1811–1815.
6. Chan, C.Y.; Wang, S.J.; Liu, I.J.; Chiu, Y.C. A simple method for determining the critical micellar concentration of a surfactant. *J. Chin. Chem. Soc.* **1987**, *34*, 243–246.
7. Mandavi, R.; Sar, S. K.; Ranthore, N. Critical micelle concentration of surfactant, mixed-surfactant and polymer by different method at room temperature and its importance. *Orient. J. Chem.* **2008**, *24*, 559–564.
8. Ghosh, S.; Krishnan, A.; Das, P. K.; Ramakrishnan, S. Determination of critical micelle concentration by hyper-Rayleigh scattering. *J. Am. Chem. Soc.* **2003**, *125*, 1602–1606.
